# Ancestral QTL Alleles from Wild Emmer Wheat Enhance Root Development under Drought in Modern Wheat

**DOI:** 10.3389/fpls.2017.00703

**Published:** 2017-05-09

**Authors:** Lianne Merchuk-Ovnat, Tzion Fahima, Jhonathan E. Ephrath, Tamar Krugman, Yehoshua Saranga

**Affiliations:** ^1^The Robert H. Smith Institute of Plant Sciences and Genetics in Agriculture, Hebrew University of JerusalemRehovot, Israel; ^2^Institute of Evolution, University of HaifaHaifa, Israel; ^3^French Associates Institute for Agriculture and Biotechnology of Drylands, Jacob Blaustein Institutes for Desert Research, Ben-Gurion University of the NegevBeersheba, Israel

**Keywords:** grain yield, near-isogenic line, quantitative trait locus, root system architecture, *Triticum turgidum* ssp. *dicoccoides*, water stress

## Abstract

A near-isogenic line (NIL-7A-B-2), introgressed with a quantitative trait locus (QTL) on chromosome 7AS from wild emmer wheat (*Triticum turgidum* ssp. *dicoccoides*) into the background of bread wheat (*T. aestivum* L.) cv. BarNir, was recently developed and studied in our lab. NIL-7A-B-2 exhibited better productivity and photosynthetic capacity than its recurrent parent across a range of environments. Here we tested the hypothesis that root-system modifications play a major role in NIL-7A-B-2’s agronomical superiority. Root-system architecture (dry matter and projected surface area) and shoot parameters of NIL-7A-B-2 and ‘BarNir’ were evaluated at 40, 62, and 82 days after planting (DAP) in a sand-tube experiment, and root tip number was assessed in a ‘cigar-roll’ seedling experiment, both under well-watered and water-limited (WL) treatments. At 82 DAP, under WL treatment, NIL-7A-B-2 presented greater investment in deep roots (depth 40–100 cm) than ‘BarNir,’ with the most pronounced effect recorded in the 60–80 cm soil depth (60 and 40% increase for root dry matter and surface area, respectively). NIL-7A-B-2 had significantly higher root-tip numbers (∼48%) per plant than ‘BarNir’ under both treatments. These results suggest that the introgression of 7AS QTL from wild emmer wheat induced a deeper root system under progressive water stress, which may enhance abiotic stress resistance and productivity of domesticated wheat.

## Introduction

Drought, the major stress factor limiting crop productivity worldwide (Boyer, 1982; Araus et al., 2008), is expected to increase due to global climate change (Wheeler and von Braun, 2013). This poses a major challenge for the improvement of crop productivity to meet the demands of a growing human population. Breeding high-yield drought-resistant cultivars of major crops, such as wheat (*Triticum* spp.), is considered a sustainable approach to meeting this challenge. The progress in wheat yield, which was considerably accelerated with the ‘green revolution,’ has slowed over the last three decades (Fischer and Edmeades, 2010; Challinor et al., 2014; Curtis and Halford, 2014).

It has been suggested that direct selection for specific root architecture could enhance wheat yield in dryland cropping regions (Tuberosa and Salvi, 2006; Wasson et al., 2012). There is high value in targeting the capture of deeper soil moisture during the grain-filling stage because: (a) it is a relatively predictable water source, and (b) it is exploited when assimilates are being directed almost exclusively to grains and greatly contribute to yield (Wasson et al., 2012). The wheat root system is comprised of embryonic seminal roots and adventitious shoot-borne roots, both of which carry lateral roots (reviewed by Satbhai et al., 2015). In the field, the deeper third of the root system consists mainly of branched roots (∼94%) and much less of axial roots (Watt et al., 2008). Therefore, the development and growth of lateral roots is a key component shaping root-system architecture. Root-hair length and density can markedly increase the root–soil interface and is expected to have a great impact on the plant’s water and nutrient acquisition (Satbhai et al., 2015). Wasson et al. (2012) suggested an approach for developing new varieties that make better use of deep-stored water, via the identification and employment of genetic diversity for superior wheat root traits (deep and highly branched roots). However, the highly complexity of root phenotyping impairs the implementation of root characteristics in plant breeding. Likewise, studies of genetic variability and QTL mapping of root traits are limited in number (Atkinson et al., 2015).

The genetic diversity in modern crop species is tremendously depleted following domestication (i.e., founder effect) and modern breeding (Tanksley and McCouch, 1997; Ladizinsky, 1998), thus making current crop germplasm vulnerable to various biotic and abiotic stresses. The untapped biodiversity in wild progenitors of crop plants was found to be a promising source for enrichment of the domesticated gene pool by reintroducing valuable wild alleles that were ‘left behind’ (Aaronsohn, 1910; Tanksley and McCouch, 1997; Feldman and Millet, 2001; Gur and Zamir, 2004). Wild emmer wheat, *Triticum turgidum* ssp. *dicoccoides* [Körn.] Thell., is the tetraploid (2*n* = 4*x* = 28; genome BBAA) progenitor of the domesticated tetraploid (2*n* = 4*x* = 28; BBAA) durum wheat [*T. turgidum* ssp. *durum* (Desf.) MacKey] and hexaploid (2*n* = 6*x* = 42; BBAADD) bread wheat (*T. aestivum* L.) (Feldman, 2001). Wild emmer wheat evolved in the Near Eastern Fertile Crescent under a wide range of ecogeographical conditions and harbors rich allelic diversity for numerous important traits, including agronomic characteristics, grain quality and resistance to biotic and abiotic stresses (recent reviews by Peng et al. (2012) and Huang et al. (2016) and references therein). Another wild wheat relative, *Agropyron elongatum*, was found a promising donor for enhanced root architecture and water stress adaptation (Placido et al., 2013). Despite its great potential, the wild wheat gene pool has not been widely exploited in wheat breeding for abiotic stress (Jaradat, 2011), possibly due to the complexity of the traits involved and the long duration of gene introgression from wild germplasm.

In previous studies, we explored the potential of wild emmer wheat to enhance drought resistance in domesticated wheat (Peleg et al., 2005) and mapped quantitative trait loci (QTLs) associated with drought-related traits (Peleg et al., 2009). Subsequently, selected QTLs in which the wild alleles showed an advantage over the domesticated alleles in productivity under drought or susceptibility indices, were introgressed via marker-assisted selection into elite durum and bread wheat cultivars (Merchuk-Ovnat et al., 2016a). Several of the resultant BC_3_F_3_ and BC_3_F_4_ near-isogenic lines (NILs) exhibited better yield and drought resistance than their recurrent parents. The NIL-7A-B-2, introgressed with the 7AS genomic region from wild emmer wheat into the background of bread wheat cv. BarNir, exhibited consistently improved grain yield and aboveground dry matter (DM), particularly under water-limited (WL) conditions, in both single-row microplots and medium- and high-density plots in a rain-protected screenhouse and open field, respectively (Merchuk-Ovnat et al., 2016a,b). The superior biomass and yield presented by NIL-7A-B-2 was accompanied by improved rate and efficiency of flag leaf photosynthesis. Soil sampling to assess gravimetrical water content at medium planting density exposed greater root branching at the margins of the drill, which led us to hypothesize that modified root architecture may be among the factors underlying the superior performance of NIL-7A-B-2. In the current study root and shoot traits of NIL-7A-B-2 and its recurrent parent, cv. BarNir, were investigated under contrasting water availabilities in controlled environments.

## Materials and Methods

### Development of NILs

A marker-assisted backcross program was employed for the introgression of the wild donor (*T. turgidum* ssp. *dicoccoides*, acc. G18-16) alleles in selected QTL regions into a widely cultivated elite Israeli bread wheat cultivar (cv.), BarNir, as described previously by Merchuk-Ovnat et al. (2016a). Briefly, marker-assisted selection was based on selected simple sequence repeats (SSRs) flanking the target QTL region. Extraction of genomic DNA was based on Doyle and Doyle (1990), PCR amplification of SSR markers followed Peleg et al. (2008), fragments were detected by genetic analyzer (3130XL, Applied Biosystems, Foster City, CA, USA) and data were analyzed with Peak Scanner Software version 2.1 (Applied Biosystems). The resultant BC_3_F_3_ NILs and their recurrent parents were genotyped using the Wheat 15K SNP array (TraitGenetics GmbH, Gatersleben, Germany^1^) containing 12,905 markers selected from the wheat 90K SNP array (Wang et al., 2014). To identify and verify the introgressed segments we aligned our SNP genotyping with the high-density tetraploid wheat consensus map (Maccaferri et al., 2015). NIL-7A-B-2, targeted in the current study, seemed to have two introgressed segments (53.1–89.8 and 116–119.7 cM), presumably due to double recombination (Table S1 in Merchuk-Ovnat et al., 2016a).

### Sand-Tube Experiment

**FIGURE 1:**
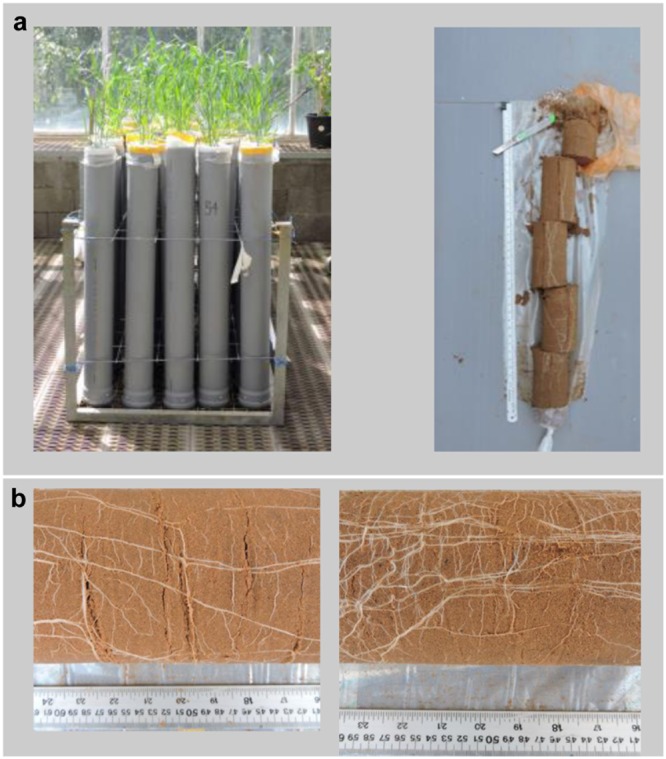
**(a)** Sand-tube experiment (left) and five 20-cm soil layers dissected from the soil core of each tube from 0 to 20 cm, 20 to 40 cm, 40 to 60 cm, 60 to 80 cm, 80 to 100 cm (right). **(b)** Sections of 40–60 cm soil depth at 62 days after planting (DAP) of ‘BarNir’ (left) and NIL-7A-B-2 (right) under the well-watered (WW) treatment.

Seeds of NIL-7A-B-2 and its recurrent parent, cv. BarNir were placed in moist germination paper for a week in a dark cold room (4°C), followed by a week of acclimation at room temperature (22°C). Two seedlings were then transplanted into a polyvinyl chloride tube (1 m height × 10 cm diameter), which was lined inside with a clear polyethylene sleeve. Holes were made at the bottom of the plastic sleeve as well as in the base of the tube to enable adequate drainage. Each tube was filled with 0.2 kg tuff gravel at the bottom to enhance drainage, 11 kg of air-dried brown-red degrading sandy loam (Rhodoxeralf), composed of 76% sand, 8% silt, and 16% clay (w/w), and 0.1 kg fine white gravel on top to minimize soil surface evaporation. To ensure a uniform soil density, bare transparent plastic sleeves were gradually filled with the dry soil while inspecting them to avoid air pockets, after which each plastic sleeve was placed in a PVC tube. The water content at field capacity was 2.5 L and the total tube weight was 15.1 kg (**Figure 1a**).

**FIGURE 2:**
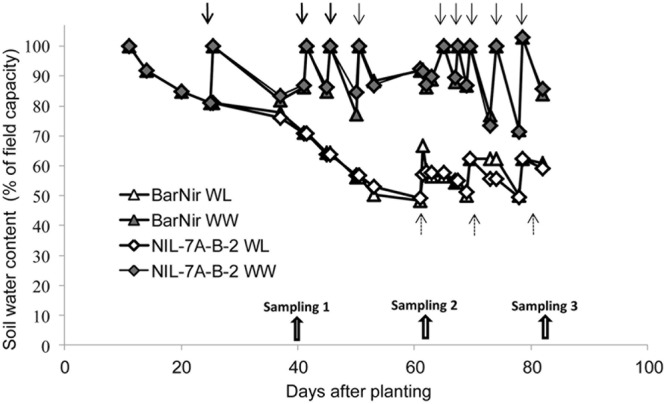
**Soil water content as a function of days from planting in the sand tubes of NIL-7A-B-2 and its recurrent parent ‘BarNir’ under the WW and water-limited (WL) treatments.** Irrigation (marked with arrows) for the WW treatment was applied when field capacity dropped to 80%, whereas for the WL treatment, irrigation was applied when field capacity was at 50%, raising it to 60%. Plants were sampled on three dates: 40, 62, and 82 DAP.

Three times weekly, tubes were manually placed on a digital floor scale and weighed to monitor their water content. Plant fresh weight at the end of the experiment was at most 2–3% of the soil water and thus it could be neglected. Irrigation for the well-watered (WW) treatment was applied when water content dropped to 80% of field capacity, raising it to 100%, whereas irrigation for the WL treatment was applied at 50% field capacity, raising it to 60% (**Figure 2**). Plants were grown in a phytotron under natural light and controlled temperatures starting with 10/16°C (night/day), and continuing with 16/22°C after 40 days (first sampling) to mimic temperature patterns under open field conditions.

**FIGURE 3:**
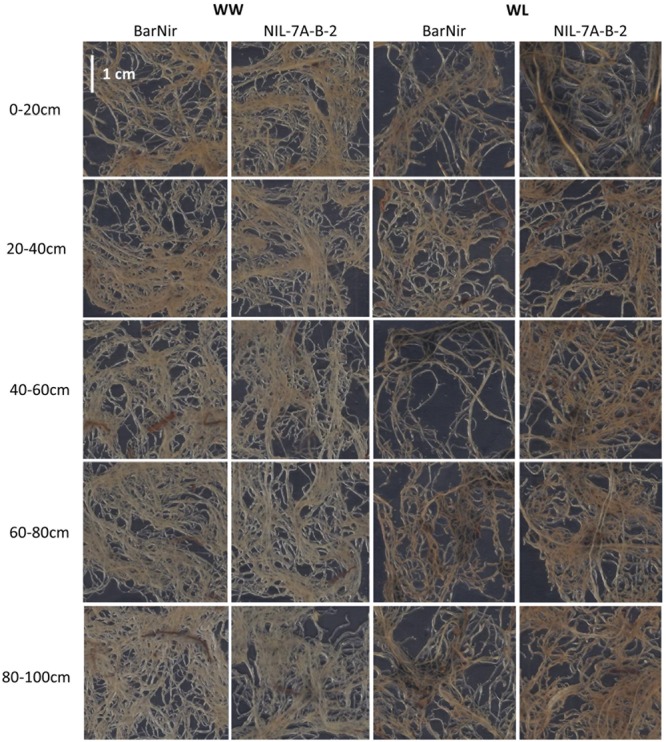
**Examples of scanned roots from each of the five sampled soil depths of ‘BarNir’ (left) and NIL-7A-B-2 (right) under the WW and WL treatments, at 82 DAP.** Sections of 3.6 × 3.6 cm were sampled from the center of ∼20 × 30 cm scanned images.

A factorial (2 genotype × 2 irrigation treatments) block design with five replicates was employed. Each experimental unit (genotype × treatment × replicate) consisted of three tubes to allow destructive samplings that were carried out at 40, 62, and 82 days after planting (DAP). On each of the sampling dates, the entire aboveground plant material from one tube per experimental unit was harvested, and the polyethylene sleeves were removed from each tube and carefully cut along the soil profile. The soil core of each tube was photographed and cut into five sections: 0–20 cm, 20–40 cm, 40–60 cm, 60–80 cm, and 80–100 cm (**Figures 1a,b**). Each root section was thoroughly washed with tap water and stored in Petri dish with some water at 4°C. Within 48 h after sampling, roots were gently spread in a water container, transferred onto a transparent film and the entire root mass of each section was scanned with a black background (**Figure 3**). Scanned root images were analyzed to determine root projected surface area (PSA) using a custom-made software (unpublished). Ten shades that covered the range of colors in the measured object area were visually sampled from the root images. Pixels ‘close’ to one of the specified colors according to a RGB metric (within user-specified tolerance) were considered part of the measured object, while pixels further away were considered background.

Gas exchange of the flag leaf was measured at 61 DAP between ∼0900 and 1200 h using a portable photosynthesis system (Li-6400, Li-COR Inc., Lincoln, NE, USA). Leaf cuvette was set at a photosynthetic photon flux density (PPFD) of 1000 μmol/m^2^s, and a temperature of 25°C. Root and shoot DM were determined after drying for 5 days at 60°C. The ratio between root and shoot DM was calculated. Culm length was measured at 62 DAP and 82 DAP, and heading day, defined as the date at which the first spikes in each experimental unit were fully exposed, were determined by daily inspection.

### Seedling Experiment

**FIGURE 4:**
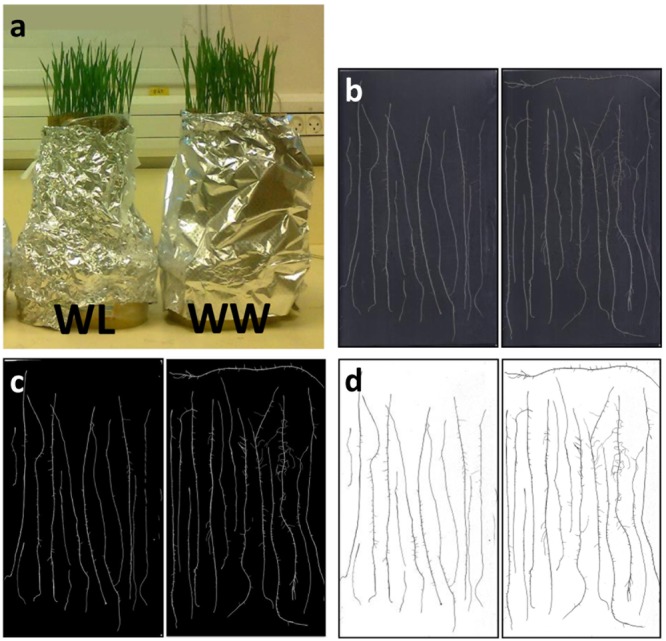
**(a)** Image of the ‘cigar-roll’ seedling experiment at 14 days after germination. Roots of three plants of cv. BarNir (left) and NIL-7A-B-2 (right; **b**) scanned on a black background at 600 dpi, **(c)** thresholded to eliminate background noise, and **(d)** inverted scans used to count number of root tips.

Seeds were placed on two layers of moist tan ‘regular weight’ seed germination paper (Anchor Paper Co., St Paul, MN, USA) in 90 mm Petri dishes and allowed to germinate in the dark for 24 h at 22 ± 1°C. Eight seeds from which the first seminal root had emerged were then placed between two (25.4 × 38.1 cm) germination papers (as specified above) soaked in double-distilled water supplemented with a broad-spectrum fungicide (15 mg/L Merpan, Captan 80% water-dispersible granule). Seeds were placed about 2.5 cm below the top of the germination paper (at ‘landscape’ orientation) and each pair of papers holding the seeds was rolled and placed in one of two 1 l flat containers, one filled with 0.5 l (∼4 cm depth) of the fungicide solution (WW) and the second without water (WL treatment), each treatment included five replicates. The containers were wrapped with aluminum foil to darken only the root environment (**Figure 4a**). The WW container was kept with 4 cm of fungicide solution, while the WL paper rolls were soaked with such solution once after 7 days and then left to dry gradually, yet remaining moist. At 14 days after germination, prior to adventitious root development, seminal roots of three random seedlings were excised and scanned over a black background at 600 dpi (**Figure 4b**). Next, the images were thresholded to eliminate background noise (**Figure 4c**) and images were inverted (**Figure 4d**) using a designated custom-made image-analysis software (unpublished). Finally, the root-tip number and root diameter were monitored by an automated image analysis, using a publicly available graphic optimization tool (*RootGraph*, Cai et al., 2015).^2^

### Statistical Analyses

The JMP version 12.0 statistical package (SAS Institute, Cary, NC, USA) was used for statistical analyses. A factorial model was employed for analysis of variance (ANOVA) for both experiments, with genotype, irrigation treatment and their interaction as fixed effects. Student’s *t*-test was employed to compare between the two genotypes averaged across treatments as well as under specific treatments. Drought susceptibility index (S) was calculated for the measured traits according to Fischer and Maurer (1978) as: *S* = (1–*Y*_WL_/*Y*_WW_)/(1–*X*_WL_/*X*_WW_), where *Y*_WL_ is a single repeat of a certain genotype under WL treatment, *Y*_WW_ is the mean performance of the same genotype under WW treatment, and *X*_WL_ and *X*_WW_ are the mean performances of both genotypes under the respective treatments.

## Results

### Sand-Tube Experiment

Near-isogenic line-7A-B-2 and its recurrent parent cv. BarNir were tested under contrasting water availabilities in a sand-tube experiment. A factorial model ANOVA carried out separately for each sampling date showed significant effects of genotype and irrigation in a number of cases with the most pronounced effect of water availability manifested at the last sampling date for all variables (**Table 1**). Gas exchange measurements at 61 DAP showed dramatic reduction of stomatal conductance under the WL treatment (*g*_s370_ = 0.08 mmol/m^2^s), compared to the WW treatment (*g*_s370_ = 0.5 mmol/m^2^s), indicating severe stress induced by the water limitation, with no major differences between genotypes. Such conductance values are considered un-acceptable for gas exchange measurements, and therefore these data were not further analyzed and are not presented.

**Table 1:** Root and shoot characteristics of NIL-7A-B-2 and its recurrent parent ‘BarNir’ under well-watered (WW) and water-limited (WL) treatments and their averages across treatments at 40, 62, and 82 days after planting (DAP).

Sampling time	Treatment	Genotype	Root PSA cm^2^/plant	Root DM mg/plant	Shoot DM mg/plant	Total DM mg/plant	Root:Shoot DM ratio
40 DAP	WW	BarNir	100.6	294	860	1154	0.33
		NIL-7A-B-2	120.4	285	921	1224	0.33
							
	WL	BarNir	95.4 (2.02)	235 (1.46)	678 (1.26)	913 (1.31)	0.35 (3.19)
		NIL-7A-B-2	119.8 (0.19)	264 (1.53)	712 (0.72)	976 (0.78)	0.37 (-0.79)
						(∗)	
	Avg	BarNir	98	264	769	1033	0.34
		NIL-7A-B-2	120.1	275	805	1086	0.35
			∗				

62 DAP	WW	BarNir	237.5	581	3376	3956	0.18
		NIL-7A-B-2	292	761	4513	5274	0.17
			∗∗	∗∗	∗∗	∗∗	
	WL	BarNir	178.1 (0.8)	506 (1.73)	2984 (0.37)	3490 (0.40)	0.18 (-0.02)
		NIL-7A-B-2	186.4 (1.16)	598 (1.21)	2405 (1.47)	3002 (1.45)	0.25 (2.12)
					(∗∗)	(∗∗)	∗∗ (∗)
	Avg	BarNir	204.5	5433	3180	3723	0.18
		NIL-7A-B-2	231.3	6794	3459	4138	0.21
			∗	∗			∗

82 DAP	WW	BarNir	306	654	9247	9901	0.07
		NIL-7A-B-2	320.8	716	10548	11264	0.07
					∗	∗	
	WL	BarNir	159.5 (1.12)	358 (1.19)	3368 (0.99)	3726 (1.00)	0.11 (0.65)
		NIL-7A-B-2	199.0 (0.89)	490 (0.83)	3738 (1.01)	4228 (1.00)	0.13 (1.37)
			∗ (∗)	∗ (∗)			∗ (∗)
	Avg	BarNir	232.7	506	6307	6814	0.09
		NIL-7A-B-2	259.9	603	7143	7746	0.1
				∗	∗	∗	

	**Source of variation**	**d.f.**	**F ratio**	**F ratio**	**F ratio**	**F ratio**	**F ratio**

40 DAP	Genotype (G)	1	10.99^∗∗^(0.33)	0.1 (3.33)	1 (3.87)	0.78 (5.34^∗^)	0.08 (0.32)
	Irrigation (I)	1	0.09	1.46	17.02^∗∗∗^	10.46^∗∗^	0.95
	G × I	1	0.87	0.34	0.08	0	0.33
	Error b (residual)	16 (8)					

62 DAP	Genotype (G)	1	7.76^∗^ (3.35)	10.1^∗∗^ (3.74)	1.15 (16.74^∗∗^)	2.1 (19.01^∗∗^)	4.51^∗^ (8.12^∗^)
	Irrigation (I)	1	53.54^∗∗∗^	7.76^∗^	23.06^∗∗∗^	22.87^∗∗∗^	6.19^∗^
	G × I	1	4.21	1.07	10.89^∗∗^	9.95^∗∗^	6.19^∗^
	Error b (residual)	16 (8)					

82 DAP	Genotype (G)	1	2.89 (11.77^∗^)	4.75^∗^ (10.43^∗^)	6.09^∗^ (0.29)	6.67^∗^ (0.00)	2.4 (9.54^∗^)
	Irrigation (I)	1	70.54^∗∗∗^	34.38^∗∗∗^	351.86^∗∗∗^	334.9^∗∗∗^	51.50^∗∗∗^
	G × I	1	0.6	0.62	1.89	1.42	5.72^∗^
	Error b (residual)	16 (8)					

*Values of root projected surface area (PSA) and dry matter (DM) are sums of the entire root profile (0–100 cm depth). ^∗^, ^∗∗^, and ^∗∗∗^ indicate significant difference between genotypes within a sampling day and irrigation treatment according to Student’s *t*-test, or significant F ratio in the ANOVA, at *P* < 0.05, 0.01, and 0.001, respectively. Susceptibility indices, and the respective significance of difference between genotypes are indicated in parentheses.*

As expected, water limitation caused a significant decrease in average root DM and shoot DM in both genotypes, and the effect appeared to become greater with each sampling date (**Table 1**). At 40 DAP, the average root DM and shoot DM under WL were reduced by 14% (not significant) and 22%, respectively, compared to the WW treatment, whereas root PSA was not affected. At 62 DAP, both root PSA and shoot DM under the WL treatment were reduced by 29% relative to the WW treatment, whereas root DM was reduced by only 18%. At the final sampling, 82 DAP, WL shoot DM was reduced by 36% relative to the WW treatment, while root DM and root PSA were reduced by 61 and 57%, respectively.

**FIGURE 5:**
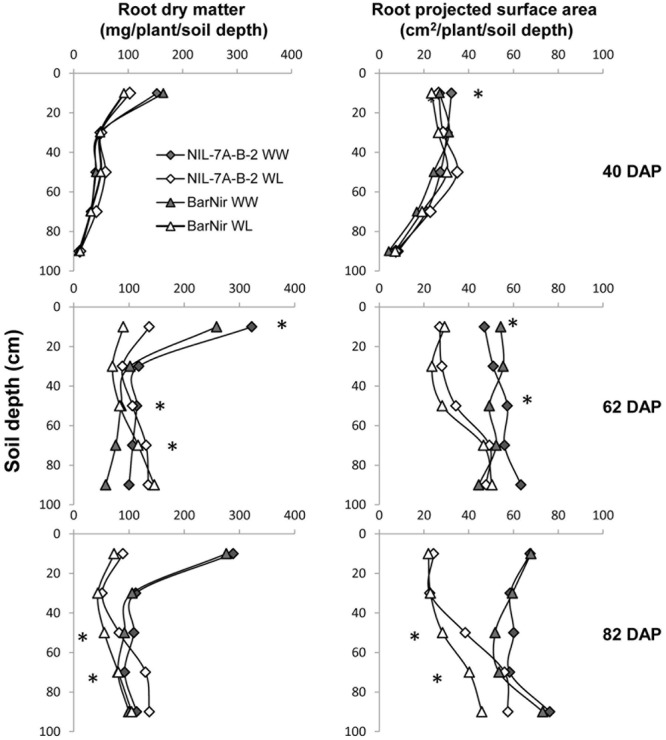
**Root dry matter (left) and projected surface area (PSA, right) across the different 20-cm soil layers under the WW and WL treatments, for NIL-7A-B-2 and ‘BarNir’ on 40, 62, and 82 DAP in the sand-tube experiment.** ^∗^Indicate significant differences between NIL-B-7A-2 and cv. BarNir. at *P* < 0.05 under WW treatment (on the right of the graph) or WL treatment (left of the graph).

A comparison between root DM of the two genotypes showed no significant difference at 40 DAP, however, at 62 DAP, under the WW treatment, NIL-7A-B-2 presented significantly higher root DM in the 40–60 cm and 80–100 cm depths, as well as for the entire profile (**Figure 5** and **Table 1**). At 82 DAP under the WL treatment, NIL-7A-B-2 presented significantly greater root DM than cv. BarNir in depths 40–60 cm and 60–80 cm (**Figure 5**), as well as for the entire profile (**Table 1**), with no significant differences under the WW treatment.

Root PSA for the entire profile and averaged across treatments showed significant differences at 40 DAP, with advantage of NIL-7A-B-2 over cv. BarNir (**Table 1**). Mixed trends were observed at 62 DAP: the 0–20 cm depth, ‘BarNir’ showed greater root PSA than NIL-7A-B-2, whereas the opposite trend was observed in the 40–60 cm depth as well as for the entire profile (**Figure 5** and **Table 1**). Notably, at 82 DAP, NIL-7A-B-2 under the WL treatment exhibited a significantly greater root PSA than its recurrent parent cv. BarNir in both depths 40–60 and 60–80 cm (**Figure 5**) and for the entire profile (*P* = 0.08, **Table 1**).

Shoot variables did not show significant differences between the two genotypes in both treatments at 40 DAP (**Table 1**). At 62 and 82 DAP under the WW treatment, NIL-7A-B-2 showed significantly higher shoot DM and total DM than cv. BarNir, as well as averaged across treatments at 82 DAP. Under the WL treatment, root-to-shoot ratio at both 62 and 82 DAP was significantly higher in NIL-7A-B-2 than cv. BarNir.

Drought susceptibility indices (S), reflecting the stability across environments, were calculated. At 40 DAP, NIL-7A-B-2 presented lower S (greater stability) for total DM than BarNir (**Table 1**). At 62 DAP, BarNir presented lower S for shoot and total DM compared to NIL-7A-B-2, which appears to be influenced by the lower productivity of the former under WW treatment. At 82 DAP, NIL-7A-B-2 presented lower *S* values than BarNir for both root PSA and root DM, while no differences between genotypes were observed for shoot and total DM. While for PSA and DM variables low *S* values reflect greater stability across environment, for Root:Shoot ratio high *S* values reflect greater plasticity across environments as manifested by NIL-7A-B-2 at both 62 and 82 DAP.

In agreement with our previous data (Merchuk-Ovnat et al., 2016a,b), NIL-7A-B-2 exhibited slightly delayed heading (58.6 vs. 55.4, averages across treatments) as compared to its recurrent parent ‘BarNir’ and higher column length (51.6 vs. 42.2, respectively; data not shown).

### Seedling Experiment

**FIGURE 6:**
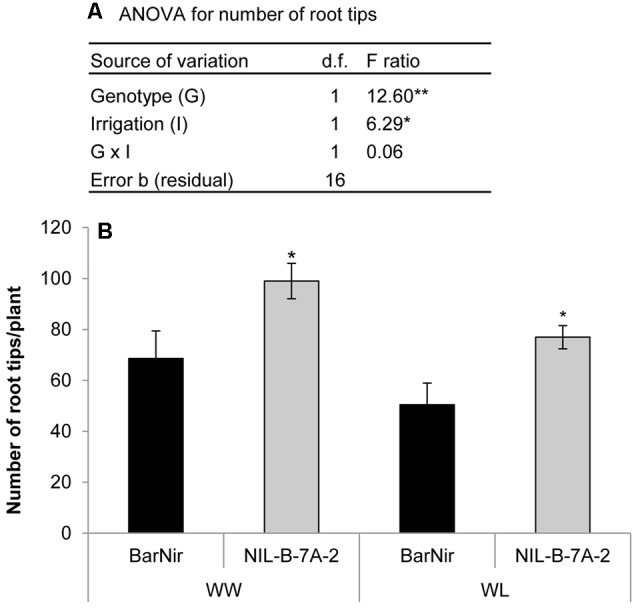
**‘Cigar-roll’ seedling experiment (A)** Analysis of variance for root-tip number, and **(B)** root-tip numbers per plant in NIL-B-7A-2 and its recurrent parent ‘BarNir’ under WW control (black bars) and WL treatment (gray bars). ^∗^ and ^∗∗^ indicate significant F ratio in the ANOVA or significant difference between genotypes within irrigation treatment (by Student *t*-test) at *P* < 0.05 and 0.01, respectively.

Seedlings of NIL-7A-B-2 and its recurrent parent cv. BarNir were grown in a germination paper (‘cigar roll’) under different water availabilities and evaluated for root-tip number, reflecting seminal root-branching capacity. A factorial model ANOVA exposed a significant effect of genotype and irrigation for root-tip number per plant (**Figure 6A**). Root-tip number under the WL treatment was ∼76% of that obtained under the WW treatment. Nevertheless, under both treatments, NIL-7A-B-2 exhibited greater root-tip number than ‘BarNir’ (44 and 52% under the WW and WL treatment, respectively, **Figure 6B**). No significant differences were found between genotypes in root diameter (data not presented).

## Discussion

Root-system characterization is highly complex, labor-intensive and time-consuming, particularly in mature plants. In wheat, and in other plant species, only a handful of studies have attempted to characterize the root system in mature plants, usually under specially designed artificial growth conditions (Kuijken et al., 2015); even fewer studies have been conducted under field conditions (Narayanan et al., 2014 and references therein). Recently developed high-throughput root-phenotyping pipelines are usually limited to seedlings which are grown hydroponically, in agarose gel or on germination papers (Atkinson et al., 2015, and references therein). Although seedling root traits have been found to be indicative of certain mature plant traits, such as N uptake (Liao et al., 2004) and drought tolerance (Manschadi et al., 2007), different sets of root-trait-influencing genes might be expressed at different stages of the plant’s life cycle (Ehdaie et al., 2014). Therefore, an assessment of root characteristics at various growth stages and under various conditions, as performed in the current study, may provide a wider view of root traits and its phenotypic dynamics.

In agreement with the difficulties involved in root-system characterization, studies of genetic variability and QTL mapping of root traits are limited in number and usually conducted at the seedling stage (Atkinson et al., 2015, and references therein), thus they are of minor relevance for mature field-grown plants. A different approach was implemented in our studies. QTLs conferring drought resistance and productivity were mapped and introgressed from wild emmer wheat into modern wheat cultivars. SNP genotyping of the resultant NILs provided a high resolution confirmation for the introgressed genomic regions and showed that between 3.3 and 9.5% of the markers were introgressed from the donor parent including the targeted genomic regions (Merchuk-Ovnat et al., 2016a), or 3.6% for NIL-7A-B-2 examined in the current study. NIL-7A-B-2, carrying the 7AS genomic region from wild emmer wheat in the background of bread wheat cv. BarNir, exhibited greater productivity, photosynthetic rate and stomatal conductance than the recurrent parent under water limitation, suggesting a more favorable water status (Merchuk-Ovnat et al., 2016a,b). These results, as well as preliminary observations of this line’s root system, led us to hypothesize that introgression of the wild emmer allele on chromosome 7AS has an effect on the root system.

**FIGURE 7:**
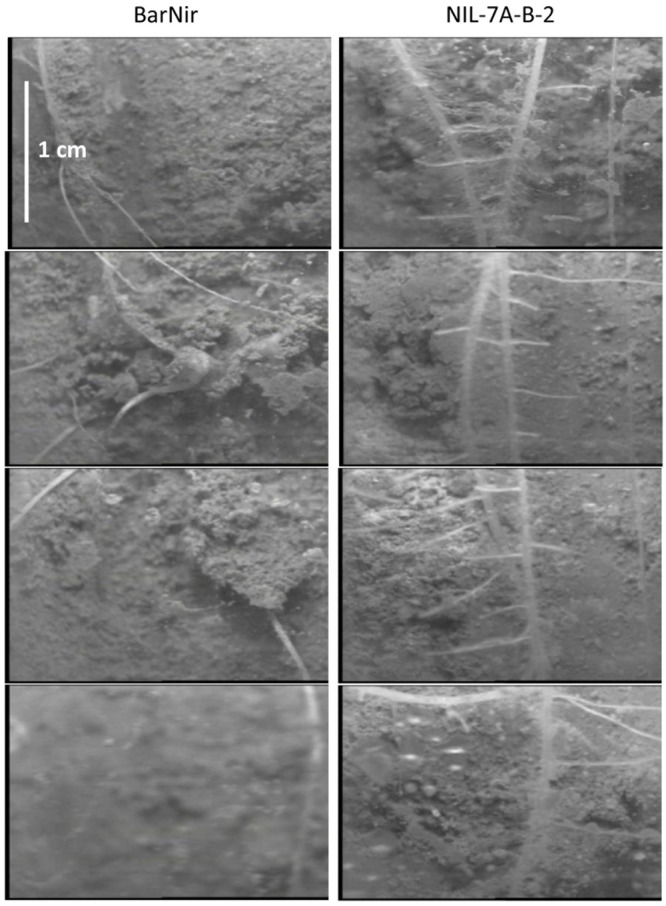
**Selected photographs taken in the field using a mini-rhizotron system at depth of 90–96 cm (1.5 cm high frames) for BarNir (left) and NIL-7A-B-2 (right).** Field trials are described in Merchuk-Ovnat et al. (2016b).

Visual observation of our sand-tube soil cores seemed to show a greater number of lateral roots in NIL-7A-B-2 compared to ‘BarNir’ (**Figure 1b**). This phenomenon was further studied and quantified in our ‘cigar-roll’ seedling experiment, which showed a significantly greater number of root tips in NIL-7A-B-2 under both treatments (**Figures 4, 6**). Mini-rhizotron observation tubes were installed in our field trials (Environments 3 and 4 in Merchuk-Ovnat et al., 2016b). However, in most cases, we failed to trace roots, presumably due to small gaps between the soil and the transparent observation tubes. Nevertheless, the few root images acquired by the mini-rhizotron, although not sufficient for statistical analysis, provide a visual evidence of a greater root density in NIL-7A-B-2 (**Figure 7**). Thus, the data recorded in our sand-tube experiment were supported by both the ‘cigar-role’ seedling experiment and mini-rhizotron observation in the field.

**FIGURE 8:**
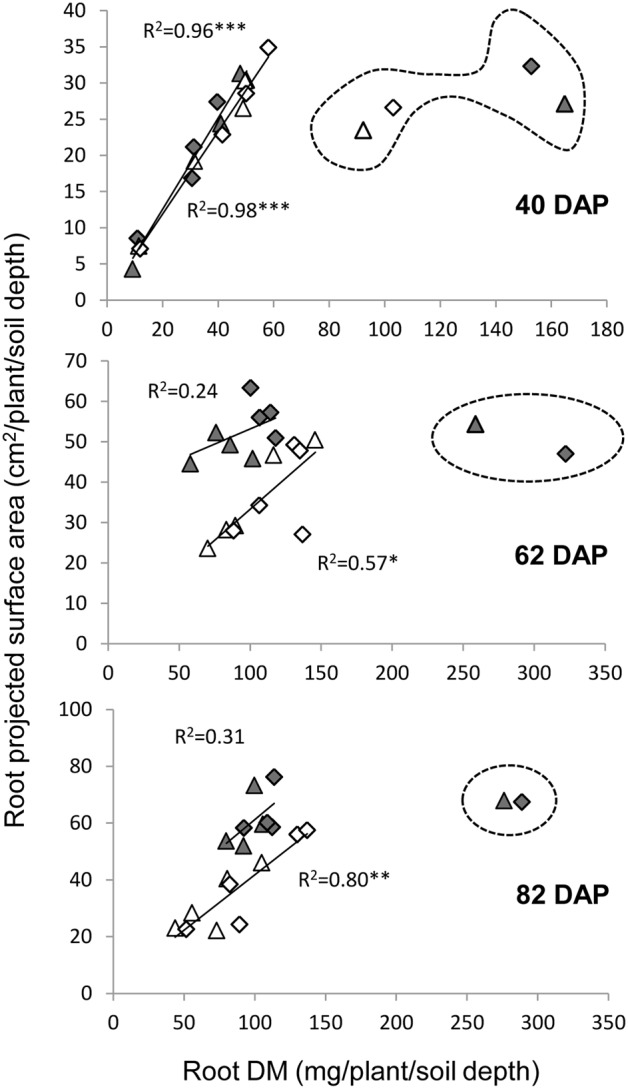
**Relationships between root dry matter and root PSA under the WW (closed symbols) and WL (open symbols) treatments on 40, 62, and 82 DAP, for the different depths and genotypes.** Data points surrounded by dashed line were excluded from the correlation analysis. ^∗^, ^∗∗^, and ^∗∗∗^ indicate significant correlation coefficient at *P* < 0.05, 0.01, and 0.001, respectively.

Two variables were used in our sand tube experiment to quantify the root system, DM and PSA. An interesting phenomenon was highlighted by the associations between root DM and PSA, in which the 0–20 cm depth clearly diverged from all other data points at 40 DAP under both treatments and at 62 and 82 DAP under the WW treatment (**Figure 8**). These data, indicating greater root weight per PSA in the upper soil layer, seemed to reflect a greater proportion of thick roots (either seminal, crown or lateral), which contribute more to DM than to PSA, as could be also observed in the scanned root images (**Figure 3**). Thicker roots in the upper soil layer are primarily transporters of water and minerals rather than absorbers (Wu et al., 2011; Vetterlein and Doussan, 2016), and therefore PSA appears to better represent the root’s absorbing capacity. After exclusion of these outlying data points, correlations between root DM and root PSA showed very high coefficients at 40 DAP for both treatments, whereas at 62 and 82 DAP, the coefficients were lower, albeit significant for the WL treatment, whereas no association was evident for the WW treatment.

The sand-tube experiment enabled tracing root-system distribution and dynamics of NIL-7A-B-2 and its recurrent parent under contrasting water availabilities until the grain development stage. The first root sampling was conducted at 40 DAP, prior to the establishment of a considerable difference in soil water content between the two treatments (**Figure 2**). At that time point, no major differences were recorded between genotypes or treatments (**Figure 5** and **Table 1**). During the next growth phase, the WW treatment received frequent irrigation to maintain its soil water content between 80 and 100% field capacity, whereas the WL treatment did not receive any irrigation and its soil water content decreased gradually, reaching its designated 50% threshold at about 62 DAP (**Figure 2**). The second sample taken at this time point showed greater root DM and PSA in NIL-7A-B-2 compared to ‘BarNir’ under the WW treatment, whereas under the WL treatment, the differences were not significant. Subsequently, frequent irrigation was maintained in the WW treatment while the WL treatment was irrigated fewer times and remained under continuous water deficit, between 50 and 60% field capacity. At the third and final sampling (82 DAP), NIL-7A-B-2 under the WL treatment exhibited greater root DM and PSA than ‘BarNir,’ particularly in the deeper soil layers (**Figure 5** and **Table 1**). Moreover, NIL-7A-B-2 also exhibited at the final sampling greater stability across environment (low S indexes) than BarNir for both root PSA and root DM, as well as greater plasticity (high *S* values) for Root:Shoot ratio. These results suggest that despite the severe stress conditions, NIL-7A-B-2 was capable to develop its root system to exploit water in the deep soil layers (**Figure 5**), whereas no root-system development was evident in ‘BarNir’ under WL treatment between 62 and 82 DAP. In this respect it should be noted that the roots found in the deep layers of the narrow sand tube will not necessarily reach the same depth under natural soil conditions. A greater osmotic adjustment capacity, as has been recorded in NIL-7A-B-2 (Merchuk-Ovnat et al., 2016a,b), is known to enhance root-system development under increasing water stress (Blum, 2009).

It has been proposed (Blum, 2009; Lopes and Reynolds, 2010; Wasson et al., 2012) that wheat varieties with a deeper root system (denser roots at depth rather than at the surface) have higher yields across a range of environments—from rainfed systems where crops rely on deep water for grain filling, to more favorable environments. A study of two wheat genotypes with different tolerance to WL environments in large soil-filled root-observation chambers demonstrated that the drought-tolerant genotype SeriM82 had a more uniform rooting pattern and greater root density at depth relative to the standard susceptible variety Hartog (Manschadi et al., 2006). In another sand-tube experiment, rye (*Secale cereale* L.) translocation line 1RS on the genetic background of bread wheat cultivar Pavon76 manifested consistently greater root DM at depth, as well as greater stability of grain yield across water availabilities (Ehdaie et al., 2012). Increasing deeper root growth would require greater allocation of carbon to the roots, which requires more green leaf area and/or increased photosynthetic capacity (Lopes and Reynolds, 2010). Zhan et al. (2015) reported that reduced lateral root branching density in maize (*Zea mays*) reduced the metabolic costs of soil exploration, thus permitting greater rooting depth and improved water acquisition from drying soil. In the current study, NIL-7A-B-2 exhibited greater root branching capacity compared to its parental cv. Bar-Nir across environments (a constitutive phenotype), however, the greatest development of roots, presumably lateral, took place primarily in deep soil layers and under water stress (**Figure 5**, an inductive phenotype). These two distinct cereal species (maize and wheat) seemed to employ different strategies for the same purpose, improving deep soil water acquisition.

## Conclusion

The capacity of NIL-7A-B-2 to develop a deep root system under progressive water stress is likely to be among the major factors contributing to its improved productivity, particularly under limited water availability (Merchuk-Ovnat et al., 2016a), whereas its improved photosynthetic capacity and greater flag leaf area (Merchuk-Ovnat et al., 2016b) may provide the extra assimilates required for the root development. NIL-7A-B-2 (as well as other NILs) is currently under multi-year study under field condition to assess its potential as a pre-breeding material and/or as commercial genotype. Furthermore, fine mapping of the 7A QTL region is ongoing in our lab with the aim of identifying the genes encoding the observed phenotypes and facilitating their implementation in wheat breeding.

## Author Contributions

LM-O student, marker assisted selection for development of near isogenic lines, phenotyping, data analysis and interpretation, manuscript preparation. TF co-principle investigator, guidance of marker assisted selection for development of near isogenic lines, manuscript review. JEE investigator, guidance of root phenotyping, manuscript review. TK co-principle investigator, guidance of marker assisted selection for development of near isogenic lines, manuscript review. YS principle investigator, coordination, supervision and guidance of marker assisted selection for development of near isogenic lines, phenotyping, data analysis and interpretation, manuscript preparation.

## Conflict of Interest Statement

The authors declare that the research was conducted in the absence of any commercial or financial relationships that could be construed as a potential conflict of interest. The reviewers SS and SM, and the handling Editor declared their shared affiliation, and the handling Editor states that the process nevertheless met the standards of a fair and objective review.

## Footnotes

1 http://www.traitgenetics.com/en/

2 http://www.plant-image-analysis.org/software/rootgraph
